# Associations Between Relative Deprivation and Life Satisfaction During the COVID-19 Lockdown: Results of Serial Mediation Analyses

**DOI:** 10.3389/fpsyg.2022.725373

**Published:** 2022-06-21

**Authors:** Junbo Chen, Jun Cao, Shuying Fu, Xuji Jia

**Affiliations:** ^1^School of Education, Anyang Normal University, Anyang, China; ^2^School of Media and Communication, Anyang Normal University, Anyang, China; ^3^Jiangsu Provincial Key Constructive Laboratory for Big Data of Psychology and Cognitive Science, Yancheng, China; ^4^Key Research Base of Humanities and Social Sciences of the Ministry of Education, Academy of Psychology and Behavior, Tianjin Normal University, Tianjin, China; ^5^Faculty of Psychology, Tianjin Normal University, Tianjin, China; ^6^Tianjin Social Science Laboratory of Students' Mental Development and Learning, Tianjin, China

**Keywords:** relative deprivation, belief in a just world, resilience, life satisfaction, serial mediation effect, COVID-19 pandemic

## Abstract

With the COVID-19 pandemic, life satisfaction among college students has become a key issue at universities and in society. The current study explores the effects of belief in a just world and resilience on the relationship between relative deprivation and life satisfaction. A total of 787 college students from universities in China completed online questionnaires. Results showed that relative deprivation was negatively correlated with life satisfaction. Belief in a just world and resilience separately mediated the relationship between relative deprivation and life satisfaction. Moreover, a serial mediating effect of belief in a just world and resilience was observed between relative deprivation and life satisfaction. These findings suggest that relative deprivation may impair individuals' beliefs in a just world. Moreover, less belief in a just world may lower resilience and consequently decrease life satisfaction. This study enriches the research field of relative deprivation theory in the context of the COVID-19 pandemic, and provides a new interpretation and intervention perspective for improving college students' life satisfaction.

## Introduction

With the outbreak of COVID-19, mankind is facing unprecedented challenges. Due to the lockdown associated with the pandemic, students stay at home, and they learn and communicate only through the Internet. People's lives have been affected to varying degrees, and their physical and mental health during the epidemic has attracted increasing attention. The epidemic does have a negative impact on our lives (Hawke et al., 2020; Liang et al., 2020; Pierce et al., 2020), but the COVID-19 crisis and related lockdown measures do not only have a negative impact (Ahrens et al., 2021; Tušl et al., 2021; Waters et al., 2021). The COVID-19 pandemic has reshaped people's cognitive attitudes, behavioral choices, values, and expectations (Birtus and Lǎzǎroiu, 2021; Rydell and Kucera, 2021; Watson and Popescu, 2021). Therefore, we conducted an on-line investigation to examine the life satisfaction of students during the COVID-19 pandemic, as well as possible influencing factors and the mechanism.

There is a wellknown Chinese adage ‘sit down to enjoy one's dinner, stand up to rail at inequity’, which suggests that although some people may live a better life than others, their total dissatisfaction may have increased (Zhang et al., 2009). This is a typical example of relative deprivation (RD), which is generally used to describe feelings of resentment stemming from a belief that one is deprived of a deserved outcome relative to some reference level (Crosby and Faye, 1976), and it is accompanied by feelings of anger and resentment (Smith et al., 2012). Research has demonstrated that relative deprivation can impair individuals' physical and mental health (Callan et al., 2015), decrease life satisfaction (Zhang et al., 2011), increase aggressive behavior (Greitemeyer and Sagioglou, 2017), and produce a number of negative psychological consequences, such as increased symptoms in physical stress (Walker and Mann, 1987). Relative deprivation arises from a comparison of oneself with other individuals in a society and usually triggers psychological reactions such as chronic stress (Wilkinson and Pickett, 2007) and justice-related responses (Smith et al., 2012) toward the perceived inequities.

Belief in a just world (BJW) refers to people's need to believe that the world they live in is a just world where people attain what they deserve (Lerner and Miller, 1978). BJW is related to individual beliefs about the pandemic (Devereux et al., 2021), and can protect an individual's mental health during the epidemic (Wang et al., 2021). People with strong belief in a just world are more motivated to perceive relative deprivation as fair than those with weak belief in a just world (Hafer and Olson, 1989). Since the definition of relative deprivation inherently includes low justice-related feelings that are correlated with low belief in a just world (Smith et al., 2012), it is presumable that relative deprivation is correlated with belief in a just world.

Resilience is defined as the ability to withstand stressful events with adequate physical and psychological functioning (Russo et al., 2012). Resilience plays a crucial role in the scientific and public health response to COVID-19 (Dvorsky et al., 2020; Xie et al., 2020). Resilience correlates negatively with stress (Cooke et al., 2013) and positively with life satisfaction (Cohn et al., 2009). However, BJW serves as a justifying strategy for promoting individual resilience, which is a positive adaptation in the face of adversity (Luthar and Cicchetti, 2000). Therefore, in this study, we aimed to examine the relationship between relative deprivation and life satisfaction among Chinese college students and to examine belief in a just world and resilience as possible psychological mediators of this relationship in the context of the COVID-19 pandemic.

Our study consists of an introduction, followed by a literature review, hypothesis, materials and methods. Results are then presented and discussed, highlighting the originality of the paper. The final section presents the theoretical and managerial implications, followed by limitations and future research perspectives.

## Literature Review

### The Relationship Between Relative Deprivation and Life Satisfaction

The concept of relative deprivation (RD) is identified as a type of subjective cognition and affective experience. It refers to the idea that some individuals or groups perceive themselves to have disadvantageous circumstances compared to corresponding reference groups, thereby leading them to experience negative emotions, such as anger and resentment. The origins of RD theory began with historic World War II American Soldier studies (Pettigrew, 2015; Smithi et al., 2018). A perception of “I'm working hard without getting the life I want” can trigger negative emotions such as sadness, envy, and depression, and it can reduce individuals' life satisfaction. However, relative deprivation is a cognitive process (Smith et al., 2012), and the prior literature shows that it emerges during social comparison (Crosby and Faye, 1976; Mummendey et al., 1999; Walker, 1999; Xiong and Ye, 2016), affecting subjective wellbeing (Schmitt et al., 2010). Relative deprivation is a result of social comparison, when people make judgment comparisons and compare different groups, they may display divergent psychological reactions. Usually a low percentage of such a population experiences greater satisfaction with their personal situations while relatively high numbers of people react with lowered life satisfaction but with a greater propensity to combat their circumstances. Therefore, the more a person thinks that he is at a disadvantage compared to others, the higher his relative deprivation and the lower his subjective wellbeing.

Psychologists perceive subjective wellbeing as a broad category that has several components, such as emotional responses (positive or negative), domain satisfaction, and life satisfaction (Diener et al., 2003). Therefore, subjective wellbeing includes both emotional experiences and cognitive evaluations (life satisfaction). Some studies have shown that socioeconomic status is a strong determining factor of satisfaction in life domains (Gtmez and Morcl, 1994), that household income has a significant effect on Chinese life satisfaction (Shu and Zhu, 2009), and that perceived personal status discrepancy has a negative correlation with life satisfaction (Zagefka and Brown, 2005).

### The Mediating Role of Resilience in the Relationship Between Relative Deprivation and Life Satisfaction

Relative deprivation often triggers anger and dissatisfaction (frustration), which can lead to aggressive behavior (Wright et al., 1999). It may also cause individuals to experience strong feelings of stress and depression, increase their risk of disease and promote adoption of risky behaviors such as smoking, alcohol abuse, binge eating, or drug abuse. However, negative life events can affect an individual's level of resilience (Rutter, 2006). The more an individual experiences stressful life events, the lower the level of resilience (Bonanno et al., 2007). Resilience is a dynamic construct that refers to positive adaptation in the face of adversity (Luthar et al., 2000) and the capacity to cope successfully with risk factors (Stewart et al., 1997). As a significant individual difference variable, resilience is also an important part of individual's psychological defense system (Xu et al., 2018). Many studies highlighted the association between resilience and life satisfaction and reported that individuals with strong resilience have qualities such as optimism, positive coping, and tenacity (Block and Kremen, 1996), that they can flexibly respond to stressful events, and that they have the ability to deal with negative emotions, which are vital to individual happiness and life satisfaction (Tugade and Fredrickson, 2004). Resilience is also regarded as a dynamic process that can weaken the influence of negative events and enable individuals to cope with adversity successfully (Olsson et al., 2003). Thus, individuals with high resilience may have an increased ability to face challenges, which in turn may lead to greater life satisfaction.

### The Mediating Role of Belief in a Just World in the Relationship Between Relative Deprivation and Life Satisfaction

Relative deprivation affects people's attitudes toward the social system (Smith et al., 2012). The perception of group discrimination and the experience of relative deprivation have a significant negative effect on an individual's belief in a just world (Birt and Dion, 1987). Individuals who have a strong belief in a just world have higher emotional regulation capabilities, which is conducive to maintaining mental health (Dalbert, 2002). Belief in a just world has a mediating role in the relationship between perceived discrimination and life satisfaction (Jia et al., 2020).

In fact, researchers have frequently shown that the more people believed in a just world, the higher their subjective wellbeing level was. Therefore, a strong BJW corresponds to better life satisfaction and less stress (Khera et al., 2014). Moreover, researchers have shown that several indicators of students' school-specific wellbeing are significantly related to BJW, and BJW is correlated with reduction of negative effects in unjust situations (Strelan and Sutton, 2011; Donat et al., 2016).

### The Relationship of Belief in a Just World and Resilience

Several studies have shown that belief in a just world is positively associated with resilience in different cultural contexts, such as in China and Pakistan (Wu et al., 2011; Riaz et al., 2015). Belief in a just world has an adaptive effect. It encourages people to interpret the world in a meaningful way. People with a strong BJW may be more inclined to take responsibility for their reaction to an event. A study of Chinese adolescents, for example, revealed that BJW are positively correlated with resilience (Dong, 2012). An Australian study also showed that BJW is positively associated with control, optimism, and resilience (Scholz and Strelan, 2021). It is clear that BJW is positively correlated with resilience among Chinese individuals.

## Hypothesis

**Figure 1:**
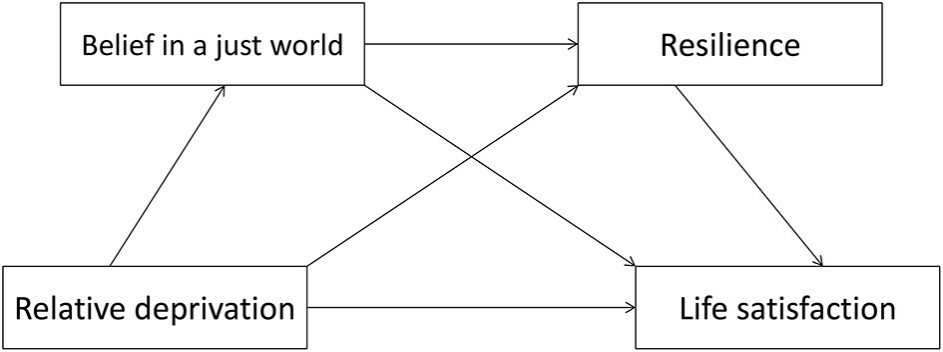
Proposed mediation model.

In this study, a serial mediation model (Figure 1) was proposed to test the mediating role of belief in a just world and resilience in the association between relative deprivation and life satisfaction. The following four hypotheses (direct and indirect effects) were examined:

Hypothesis 1: Relative deprivation is directly associated with life satisfaction.

Hypothesis 2: Relative deprivation is indirectly associated with life satisfaction via belief in a just world.

Hypothesis 3: Relative deprivation is indirectly associated with life satisfaction via resilience.

Hypothesis 4: Relative deprivation is indirectly associated with life satisfaction via belief in a just world and then resilience.

## Materials and Methods

### Participants

In this study, there were 787 respondents; 134 (17%) were male, and 653 (83%) were female. All 787 participants were Chinese college students. They were surveyed by using Questionnaire Star during the COVID-19 pandemic and the early 2020 lockdown. The age of the participants ranged between 18 and 29 years old, with an average age of 21.57 years (SD = 2.70), and 17.00% were male. They were recruited from different levels: 633 were undergraduates, and 154 were master's students.

### Instruments

#### Perceived Relative Deprivation

Two items measured perceived relative deprivation (Zagefka et al., 2013; Xiong and Ye, 2016). Participants were asked to consider their overall financial situation. The questionnaire items were “How do you think you are doing financially” (“1 = not at all well” to “5 = very well”) and ‘How satisfied are you with your financial situation’ (“1 = not at all satisfied” to “5 = very satisfied”). Items were reversed and then summed to produce composite score. Higher scores indicate higher level of perceived deprivation. The Cronbach's α coefficient of relative deprivation was 0.76.

#### Belief in a Just World

The Just World Belief Scale is a 13-item questionnaire assessing the level of belief in a just world (Dalbert, 1999). The Chinese version was translated from the English version with ensured fluency and accuracy (items were translated by the authors and back-translated by two expert English-Chinese interpreters independent of the current study) (Shu et al., 2012). Participants were required to respond on a 6-point Likert scale (1 = strongly disagree, 6 = strongly agree) to indicate the extent to which they agreed with the statements. All items were summed to produce a total score of BJW. In the current study, Cronbach's α coefficient of BJW scale was 0.92.

#### Resilience

Connor-Davidson Resilience Scale (CDRISC) was refined from the original version by Connor and Davidson (2003), and the Chinese version was developed by Yu and Zhang (2007). It consisted of 25 items (e.g., “I tend to bounce back after illness or hardship”). Respondents rated items on a 5-point Likert scale from 1 (not true at all) to 5 (true nearly all the time). The reliability coefficient of the Chinese version of the CDRISC was 0.91 (Yu and Zhang, 2007). Higher summed scores across items indicate higher level of esilience. In the assement, the reliability coefficient (Cronbach's a) of the CDRISC was 0.93.

#### Life Satisfaction

The Satisfaction with Life Scale (SWLS) is a 5-item questionnaire (Pavot and Diener, 1993) that has been applied worldwide to assess global life satisfaction (e.g., ‘So far I have gotten the important things I want in my life’). The Chinese version has good reliability and validity (Xiong and Xu, 2009). Each item is scored from 1 (strongly disagree) to 7 (strongly agree). The items were summed to determine the composite score. Higher score indicate higher level of life satifaction. For the present sample, the internal consistency of SWLS was satisfactory (Cronbach's α = 0.84).

#### Procedure

All participants were surveyed by using Questionnaire Star from March 2020 to April 2020, when the epidemic was basically under controll in China. The survey link was sent to the students' phones. All questionnaires were included in the analysis after a quality audit, with an effective rate of 100.0%. Because the study involved human participants, it was reviewed and approved by the Academic Committee of the Faculty of Psychology, Tianjin Normal University. The participants provided their written informed consent to participate in this study. Written informed consent was obtained from the individual(s) for the publication of any potentially identifiable images or data included in this article.

### Statistical Analyses

The statistical analyses were conducted using the Statistical Package for the Social Sciences (SPSS, version 26.0). Descriptive statistics were computed for demographic data and all study variables. The associations between variables were assessed by Pearson's bivariate correlation. For testing mediating effects, the bias-corrected bootstrap method provides the most accurate confidence interval (CI) estimation and has the highest statistical efficacy (Fang et al., 2012). Therefore, in the current study, a bootstrapping analysis was conducted using the SPSS macro PROCESS Model 6 (with relative deprivation as the independent variable, life satisfaction as the outcome variable, belief in a just world and resilience as mediators) with 5,000 resamples to test a serial mediation model and to calculate the 95% CIs. The indirect effect was considered statistically significant if the 95% bias-corrected CI did not contain zero (Hayes, 2013).

In addition, because the timing in the assessment of relative deprivation and life satisfaction overlapped, the mediation models were also conducted with the positions of relative deprivation and life satisfaction reversed (with life satisfaction as the independent variable, relative deprivation as the outcome variable, resilience and belief in a just world as mediators).

## Results

### Descriptive and Pearson's Correlation Results

The descriptive statistics and Pearson's correlations for all of the assessed variables are presented in Table 1. Specifically, life satisfaction was negatively and strongly associated with relative deprivation (r = −0.46, *p* < 0.001) and positively correlated with resilience (r = 0.48, *p* < 0.01). Likewise, a negative and strong relationship was also observed between relative deprivation and resilience (r = −0.20, *p* < 0.001). In addition, belief in a just world was positively and moderately correlated with life satisfaction (r = 0.45, *p* < 0.001) and negatively correlated with relative deprivation (r = −0.22, *p* < 0.001). Belief in a just world was positively and strongly correlated with resilience (r = 0.49, *p* < 0.001).

**Table 1:** Pearson's correlations among the study variables.

	**M**	**SD**	**1**	**2**	**3**	**4**
1 Relative deprivation	6.45	1.38	1.00			
2 Belief in a just world	56.60	9.75	−0.22^***^	1.00		
3 Resilience	90.25	13.00	−0.20^***^	0.49^***^	1.00	
4 Life satisfaction	20.47	5.21	−0.46^***^	0.45^***^	0.48^***^	1.00

*** *p < 0.001*.

### Testing for a Serial Mediation Model

**Figure 2:**
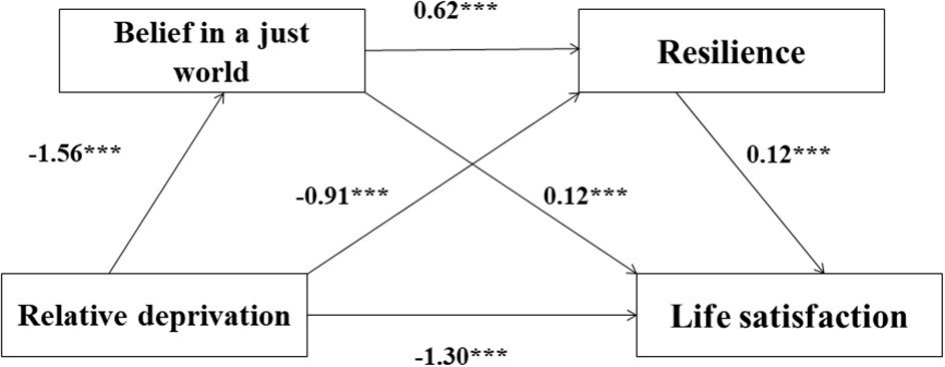
The serial mediation model showed the effects of relative deprivation, belief in a just world, and resilience on life satisfaction. *N* = 787. Regression coefficients were obtained in PROCESS Procedure for SPSS. ***p < 0.001.

We tested a serial mediation model, which consisted of three indirect effects, as follows: (1) relative deprivation enhances life satisfaction via belief in a just world, (2) relative deprivation enhances life satisfaction via resilience, and (3) relative deprivation enhances life satisfaction via belief in a just world and then resilience (Figure 2).

The results showed a negative effect of relative deprivation on belief in a just world, *B* = −1.56, *t* = −6.32, *p* < 0.001, and a negative effect of relative deprivation on resilience, B = −0.91, *t* = −3.04, *p* < 0.001. There was a positive relationship between belief in a just world and resilience, B = 0.62, *t* = 14.72, *p* < 0.001. Moreover, both belief in a just world and resilience significantly predicted life satisfaction (B = 0.12, *t* = 7.25, *p* < 0.001 for belief in a just world and B = 0.12, *t* = 9.48, *p* < 0.001 for resilience) (Table 2).

**Table 2:** Model results.

**Variables**	**Model 1 (BJW as outcome)**			**Model 2 (RE as outcome)**			**Model 3 (LF as outcome)**		
	**B**	**SE**	***t***	**B**	**SE**	***t***	**B**	**SE**	***t***
RD	−1.56	0.25	−6.32^***^	−0.91	0.30	−3.04^***^	−1.30	0.11	−12.15^***^
BJW				0.62	0.04	14.72^***^	0.12	0.02	7.25^***^
RE							0.12	0.01	9.48^***^
*R^2^*	0.05			0.25			0.41		
*F*	39.92^***^			129.07^***^			179.14^***^		

*** *p < 0.001, RD, relative deprivation; BJW, belief in a just world; RE, resilience; LS, life satisfaction*.

The results of bootstrap method for indirect effects were shown in Table 3. Indirect effect of relative deprivation on life satisfaction through belief in a just world was significant, effect = −0.19, 95% CI (−0.28, −0.11), which accounted for 11.05% of the total effect. Additionally, resilience mediated the relationship between relative deprivation and life satisfaction, effect = −0.11, 95% CI (−0.19, −0.04), which accounted for 6.40% of the total effect. Finally, the indirect effect of relative deprivation on life satisfaction through belief in a just world and then resilience (i.e., a serial mediating effect) was also found, effect = −0.12, 95% CI (−0.17, −0.07), which accounted for 6.98% of the total effect.

**Table 3:** Indirect effects of relative deprivation on life satisfaction.

	**Effect**	**Boot SE**	**Boot LL CI**	**Boot UL CI**	**Effect size**
Total indirect effect	−0.42	0.07	−0.56	−0.28	24.42%
RD → BJW → LS	−0.19	0.04	−0.28	−0.11	11.05%
RD → RE → LS	−0.11	0.04	−0.19	−0.04	6.40%
RD → BJW → RE → LS	−0.12	0.03	−0.17	−0.07	6.98%
Total effect	−1.72	0.12	−1.96	−1.49	

*RD, relative deprivation; BJW, belief in a just world; RE, resilience; LS, life satisfaction. LLCI, lower limit confidence interval; ULCI, upper limit confidence interval*.

*Effect size, effect/total effect*.

Moreover, we established two alternative models to further confirm the hypothezied model. Alternative model 1 was reversal of the mediation models (life satisfaction → resilience → belief in a just world → relative deprivation). The indirect effect of life satisfaction on relative deprivation via resilience and belief in a just world was insignificant. Alternative model 2 was reversal of two mediating variables, eg., relative deprivation → resilience → belief in a just world → life satisfaction. The alternative model 2 generated the same results as the hypothesized model. This outcome was expected because the model is essentially equivalent to the hypothesised model. Given that the hypothesized model has a stronger theoretical basis, we therefore regarded the hypothesised model as our final model.

## Discussion

The aim of our study was to investigate the relations among relative deprivation, belief in a just world, resilience and life satisfaction, and test the serial mediating effects of belief in a just world and resilience. The results of this study suggested that the mediating effects of belief in a just world and resilience may contribute to understanding the relationship between relative deprivation and life satisfaction in a sample of Chinese students.

Consistent with previous research (Zagefka and Brown, 2005; Shu and Zhu, 2009), we found that relative deprivation was a significant predictor of life satisfaction. This provided additional evidence for the negative effect of relative deprivation on adolescent life satisfaction. Some studies have showen that relative deprivation significantly increased mental illness, eroded self-rated health, and undermined life satisfaction (Zhang et al., 2011; Xia and Ma, 2020). Individuals perceived more level of relative deprivation as a stressor, indicating more unmet needs for individuals for various reasons. The degree to which an individual's needs were met was positively associated with perception of favorable life satisfaction (Sheldon et al., 2001). Together, these observations suggest that the stronger the relative deprivation felt by the students, the lower the life satisfaction they experience is.

Moreover, our results supported our second hypothesis that belief in a just world represents a potential underlying mechanism that could partially explain how relative deprivation is linked with adolescent life satisfaction. Studies on socially disadvantaged groups and on the general public indicate that belief in a just world promotes mental health (Dalbert, 2002; Furnham, 2003; Khera et al., 2014; Wang et al., 2021). Belief in a just world is particularly important for disadvantaged people (Donat et al., 2016). Relative deprivation reflects the differences in resources; students felt that these differences could lead directly to the students being at a disadvantage in future competition, resulting in psychological stress. However, this difference may also affect their mental health via their belief in a just world. Belief in a just world reflects the degree to which students believe that a disadvantage can be changed through their own efforts. If they believe that they can change the status quo through hard work, their life satisfaction is improved, they have confidence in the future, and are willing to pursue long-term goals (Dalbert, 2001). Consistent with existing research (Otto and Dalbert, 2005), although students in the pandemic may feel a stronger sense of relative deprivation, this study found that belief in a just world can serve as a personal resource for maintaining mental health. Belief in a just world appears to act as a psychological resource through its assimilation and trust functions (Dalbert and Stoeber, 2006; Dalbert and Donat, 2015). Due to these functions, people with a high belief in a just world level spend less energy on defensive behavior, and, as a result, are less anxious and healthier than those with weak belief in a just world. As a consequence of these adaptive functions, BJW has been found to be positively related to subjective wellbeing in many studies (Otto et al., 2006; Otto and Schmidt, 2007; Strelan and Sutton, 2011; Donat et al., 2016).

In previous studies (Luthar and Cicchetti, 2000; Wagstaff et al., 2018; Xu et al., 2018), a mediating role of resilience on the association between relative deprivation and life satisfaction was identified. Studies have shown that psychological resilience can positively predict life satisfaction (Abolghasemi and Varaniyab, 2010) and mental health (Ho et al., 2015). Psychological resilience reduces the effect of COVID-19 on stress and increases happiness levels (Peker and Cengiz, 2021). In addition, resilience mitigates the effect of coronavirus fear on depression, anxiety, and stress (Yildirim et al., 2020). Therefore, resilience has been found to be a protective factor of quality of life and can help students improve their mental health and life satisfaction. Students with a high level of resilience are more likely to have confidence in dealing with adversity and challenges and to be able to cope with difficulties; they tend to have a positive self-perception of mental wellbeing. Resilience plays an important role, as it is a potential means by which to facilitate the achievement of students who are disadvantaged or who are facing challenging situations. Therefore, psychological resilience can help students cope with the negative emotions brought about by relative deprivation, such as anger, dissatisfaction, disappointment, and stress, and reduce the risky behaviors caused by this, promoting students' physical and mental health.

Finally, for the first time, we found data to support the serial mediation model. Relative deprivation was indirectly associated with adolescent life satisfaction through belief in a just world and then resilience. The direct effect of BJW on resilience was significant and positive. Other evidence has also shown that BJW significantly predicts resilience (Wu et al., 2011). People with strong belief in a just world are motivated to perceive relative deprivation as fair compared to those with weak belief in a just world (Hafer and Olson, 1989). BJW enables individuals to assimilate unfair events into their just world framework, it encourages trust in the future, and it motivates people to act in a just manner (Dalbert, 2001).

In accordance with previous studies (Campbell-Sills and Stein, 2007; Wu et al., 2011), belief in a just world was positively correlated with resilience; the more strongly the participants endorsed BJW, the stronger their resilience was, and resilience was also significantly related to all indicators of subjective wellbeing. An important finding in the current study is that BJW predicts life satisfaction and independently predicts psychological resilience. This implies that the adaptive function of BJW serves a very important function and independently promotes psychological resilience to help individuals face adversities such as disasters.

Additionally, we found that the path from relative deprivation to life satisfaction via resilience and belief in a just world (an alternative model) also worked in our data. Considering that the hypothesized model has a stronger theoretical basis, we therefore selected the hypothesised model as our final model. However, we cannot denied the alternative model in our data, which need to be explored in the future research.

## Conclusion

The study from the perspective of relative deprivation theory highlighted the importance of belief in a just world and resilience in promoting college students' life satisfaction in the context of the COVID-19 pandemic. This study discovered the mediating roles of belief in a just world and resilience between relative deprivation and life satisfaction. It also found that serial mediation existed in the above relations in the Chinese culture and particular context of the COVID-19 pandemic. The findings support that it is people's cognitive beliefs that enable them to overcome negative conditions and explore negative experiences by themselves, regardless of a harsh situation.

From a managerial lens, the paper clearly highlights the fact that relative deprivation significantly undermines life satisfaction in the context of the pandemic. Therefore, colleges and universities should actively respond to the pandemic, ease the intensity of students' perception of the incident, and strengthen the shaping of students' belief in a just world and resilience, so as to improve students' life satisfaction. This study provides a reference for education management in other countries in the context of the COVID-19 pandemic.

Certain limitations of this study should be recognized. The main limitation was that our study used a cross-sectional design, which cannot provide strong evidence for causality. Although we established two alternative models to confirm the validity of the hypothesized model, it will be difficult to parse out the directionality of the relations and/or remove the influence of unmeasured confounds. Thus, further research should use a longitudinal design or experimental design to explore the realtions. Secondly, two-item measure was employed for relative deprivation. Measures with only one or a few items may suffered from unreliability and was likely to result in the masking of true effects or the weakening of the pattern of associations. Thirdly, it was worth noting that indirect effects were small, given that they are multiplicative terms and will be smaller than their constituent effects. Fourthly, because of the limited sample size and gender imbalance, these findings may not represent the entire college students. Therefore, a larger and gender-balanced sample may be required. Finally, the concept of life satisfaction is to general used. Future research could further discuss job satisfaction (Nemteanu and Dabija, 2021), and satisfaction with teleworking during the COVID-19 pandemic (Nemteanu et al., 2021).

## Data Availability Statement

The raw data supporting the conclusions of this article will be made available by the corresponding authors, without undue reservation.

## Ethics Statement

The studies involving human participants were reviewed and approved by the Academic Committee of the Faculty of Psychology, Tianjin Normal University. The patients/participants provided their written informed consent to participate in this study.

## Author Contributions

XJ conceived, designed the study, and supervised the collection of data. SF revised the manuscript and analyzed the data. JCh and JCa analysed, interpreted the data, and produced the drafting of the manuscripts. All authors listed have made direct and intellectual contribution to the article and approved the final version for publication.

## Funding

This study was funded by the Research Project on Curriculum Reform of Teacher Education in Henan Province in 2021 (Grant Number 2021-JSJYYB-038), Scientific Research Project of Tianjin Education Commission (Grant Number 2017SK142), and Jiangsu Provincial Key Constructive Laboratory for Big Data of Psychology and Cognitive Science (Grant Number 72592162001G).

## Conflict of Interest

The authors declare that the research was conducted in the absence of any commercial or financial relationships that could be construed as a potential conflict of interest.

## Publisher's Note

All claims expressed in this article are solely those of the authors and do not necessarily represent those of their affiliated organizations, or those of the publisher, the editors and the reviewers. Any product that may be evaluated in this article, or claim that may be made by its manufacturer, is not guaranteed or endorsed by the publisher.
